# Notes for Contributors

**DOI:** 10.1155/S1463924681000138

**Published:** 1981


### Viscometer with automatic density compensation

In the Nametre Model 7.010 vibrating sphere viscometer density compensation is obtained by a ten-turn counting dial that can be locked in the set position. The standard dial can be set from 0.400 to 1.400 density values. By dialling any density between the above values the electronic control unit will automatically make the division and display the result in centipoise or centistoke units. Should the compensator dial be accidentally offset from the 1.000 position by ± 0.1 turn then a red warning light bar marked "DENSITY" on the display will indicate that the density compensation computer circuit has been activated. The viscometer displays viscosity in six linearised bands in the range $10^{-1}$ to $10^5$ cp.

*Nametre Company, 1778 State Highway 27, Edison, NJ 08817, USA.*

### Multi-balance interfacing

A programmable multi-balance interface unit from Telmoss connects up to five electronic balances to a computer or display/printer terminals using two RS232C or 20 mA current loop outlets. The unit is microprocessor based and is compatible with balances from the Sartorius MP range and with the Mettler PL or PK series. The balances can be of several types or all the same. This unit is applicable for production and quality control in any industry which monitors weights, either on intermediates or on finished products. It has the flexibility to allow many types of systems to be assembled with programs tailored to individual requirements. A number of the interface units can be connected either in radial or daisy-chain configuration.

*Telmoss Ltd, 28 Dellcott Close, Welwyn Garden City, Herts, UK.*

### Automatic dissolution monitoring

The versatility of the Pye Unicam SP-100/150 series spectrophotometers has been extended by the introduction of an automatic six cell dissolution accessory. The system conforms to all current regulatory procedures in the pharmaceutical industry. It allows discrete monitoring of up to six samples against a standard or reference. Measurement intervals of 30 seconds to 99 minutes can be conveniently selected and, if required, multi-wavelength measurements and superimposed traces can be obtained on the built-in recorder. The addition of an HP97S programmable calculator allows presentation of analytical results directly in concentration with sample number. Alternatively, on input of dissolution volume, results will be printed as weight of active component dissolved. Although designed for the pharmaceutical industry and tablet dissolution applications in mind, the system is readily adaptable to any dissolution requirement; e.g. confectionery, food, agricultral or chemical industries in both quality control and research applications.

*Pye Univam Ltd, York Street, Cambridge, CB1 2PX, UK.*

### Luminescence photometer

The Turner Designs Model 20 luminescence photometer from Techmation is a microprocessor-controlled instrument for both bio and chemi-luminescence analyses. Typical applications include quantification of adenosine triphosphate for biomass determinations in marine and fresh water, control of activated sewage sludge and determination of cell viabilities in a variety of environmental, agricultural and clinical fields. The Model 20 uses a through-period optical system which includes a stabilised light source and computer-controlled shutter. This gives a system which is not only stable but also requires the minimum of day-to-day adjustments. To protect the photomultiplier from light overload the shutter closes within 50 milliseconds of light entering the instrument. This feature also simplifies sample or reagent introduction procedures. Fully selectable delay and assay times allow measurements by either peak height or integrate methods.

*Techmation Ltd, 58 Edgware Way, Edgware, Middx, HA8 8JP, UK.*

### Notes for Contributors

### Presentation of manuscripts

Manuscripts should be typed (double-spaced) on one side of the paper only and with generous margins. The title should be brief and informative avoiding the word "new" and its synonyms. The full list of authors with their affiliations and full address(es) should appear on the title page. On a separate sheet an abstract of no more than 150 words is required. This should succinctly describe the scope of the contribution and highlight significant findings or innovations. It should be written in a style which can easily be translated into French and German.

*The Concise Oxford Dictionary* and Fowler's *Modern English Usage* (both published by Oxford University Press) should be used as the standard for spelling and grammar. Abbreviations should be limited to those generally recognised, or where a frequently occuring term is abbreviated it should, in the first instance, be explained thus "flow injection analysis (FIA) ..." and the abbreviation used thereafter. Abbreviations, for standard measures and units should follow SI recommendations. There are various publications giving guidance on the use of SI units.

References should be indicated in the text by numerals following the author's name, i.e. Skeggs [6]. On a separate sheet of paper, list all references in numerical order thus:

[6] Skeggs, L.T., *American Journal of Clinical Pathology*, 1959, **28**, 311.

Note that journal titles are given in full. Where there is more than one author, the form Foreman et al. should be used in the text but all authors should be named in the list of references. When reference is made to a chapter in a book the reference should take the following form:

[7] Malmstadt, H.V. in "Topics in Automatic Chemistry" Ed. Stockwell P.B. and Foreman J.K. 1978 Horwood, Chichester, pp. 68-70.

Only work which has been published or has been accepted for publication should be cited. Avoid the citation of documents which are subject to restricted circulation, patent literature, unpublished work and personal communications. The latter can be mentioned in the text in parenthesis.

To illustrate a paper line diagrams are preferred to photographs. Photographs should only be used when they significantly add to the discussion. Diagrams, charts and graphs should be carefully drawn in black ink on stout card or heavy quality tracing paper. Most illustrations are reduced for publication; to allow for this originals should be between 16 and 36 cm wide (the depth must not exceed 50 cm). The lettering of diagrams should be sufficiently clear to withstand reduction. Except in the case of proper names, all lettering should be in lower case print. If photographs are used they must be supplied in the form of clear, unmounted, glossy, black and white prints. "Instant" photographs are not normally acceptable. All illustrations must be identified on the reverse showing the figure number and the author's name.

Each illustration should have a fully explanitory caption. Captions should be typed together on a separate sheet of paper; they must *not* be an inseparable part of the illustration.

